# Quality Improvement Project for Menstrual Health on Inpatient Psychiatric Wards

**DOI:** 10.1192/bjo.2026.11522

**Published:** 2026-06-30

**Authors:** Keonwoo Yi, Daniel Zahedi, Vinze Mullet, Pearl Gowasmi

**Affiliations:** SLAM, London, United Kingdom

## Abstract

**Aims:**

Menstrual and menopausal health significantly influences mental health, with hormonal fluctuations affecting mood regulation, emotional stability, cognition, and vulnerability to psychiatric relapse. Psychiatric medications, especially antipsychotics associated with hyperprolactinaemia, can further disrupt menstrual cycles, contributing to distress, reduced quality of life, and treatment non-adherence. Despite this, menstrual health assessment is often absent from routine psychiatric review. Systematic screening during admission is therefore essential to identify treatable contributors to mental state deterioration and to deliver truly holistic care. Our aim was to evaluate how frequently menstrual and menopausal health was assessed on all inpatient psychiatric female wards at Lambeth Hospital (South London and Maudsley Trust), and to measure the impact of targeted interventions on improving active screening rates.

**Methods:**

Baseline audit: review of clinical documentation for all patients admitted during the audit period on the wards was reviewed. Screening was categorised as:

**Active screening:** clinicians directly asked about menstrual or menopausal status and symptoms.

**Incidental screening:** information volunteered or noted indirectly.

Interventions included staff training, patient information materials, and updated clerking prompts.

A reaudit 5 months later assessed the same parameters.

**Results:**

Baseline:

- 3/44 (7%) patients actively screened.
- 8/44 (18%) patients had appropriate menstrual health intervention.
- Menstrual status was unknown for the majority of patients, and menstrual-related interventions were rare.

Re-audit:

- 28/45 (62%) patients actively screened.
- 33/45 (73%)patients had appropriate menstrual health intervention.

This represents a significant improvement on both wards. Menstrual symptoms such as dysmenorrhoea, menorrhagia, peri-menopausal changes, and amenorrhoea were more consistently identified, leading to appropriate interventions. Interventions included appropriate counselling, products and patient information leaflet.

**Conclusion:**

Our project shows that routine menstrual and menopausal health screening in psychiatric inpatient care is feasible, clinically meaningful, and substantially improved through simple service-level interventions. Embedding structured questions into admission processes enhances safe prescribing and promotes person-centred care. This project demonstrates that menstrual health assessment should be a standard component of psychiatric inpatient practice.

